# PP34 Cost Utility Of Lenalidomide With Rituximab For Follicular Lymphoma In Previously Treated Patients: The Brazilian Healthcare System Perspective

**DOI:** 10.1017/S0266462325102535

**Published:** 2025-12-29

**Authors:** Ludmila Gargano, Layssa Oliveira, Haliton Oliveira Junior, Rosa Camila Lucchetta

## Abstract

**Introduction:**

In Brazil’s Unified Health System (SUS), initial treatment for follicular lymphoma (FL) is generally effective, but many patients relapse and continue treatment with rituximab plus chemotherapy. Lenalidomide plus rituximab (LR) has been shown to improve overall survival (OS) and progression-free survival (PFS) in patients with FL. We assessed the cost utility and budget impact of LR for previously treated patients with FL from the SUS perspective.

**Methods:**

A three-state partitioned survival model was built using rituximab OS and PFS curves from the AUGMENT clinical trial, which were extrapolated over a 40-year horizon using parametric distributions and adjusted with hazard ratios (HRs) from the comparison with LR (OS: HR 0.45, 95% confidence interval [CI]: 0.22, 0.92; PFS: HR 0.40, 95% CI: 0.29, 0.56). Curve selection was based on the Akaike and Bayesian information criterion and visual inspection. Utility values were obtained from international literature. Direct medical costs, including the cost of drugs, monitoring, and pre- and post-progression were considered. For the budget impact analysis, eligible patients were estimated based on measured demand, and two different market shares were compared with the reference scenario (without LR).

**Results:**

LR showed an incremental benefit (3.10 quality-adjusted life years [QALYs]) and incremental costs (USD76,718 [BRL187,192]). The incremental cost-effectiveness ratio (ICER) suggested that LR was cost effective (USD24,712 [BRL60,298]/QALY), considering the Brazilian threshold of USD49,180 (BRL120,000) per QALY. In sensitivity analyses, LR remained cost effective in most scenarios except when the OS HR was adjusted to the upper limit of the 95 percent CI. Budget impact analysis showed that listing LR in the SUS would increase the budget by USD12,523,280 (BRL30,557,046) in five years if market share reached 50 percent by the fifth year (approximately 648 to 737 eligible patients per year).

**Conclusions:**

Standard treatment options for relapsed FL are limited. Previously treated individuals with FL may benefit more from LR than from standard treatment with rituximab and chemotherapy. The ICER showed that lenalidomide could be cost effective for the Brazilian health system.

